# Next generation sequencing techniques in neurological diseases: redefining clinical and molecular associations

**DOI:** 10.1093/hmg/ddu203

**Published:** 2014-05-02

**Authors:** Rita Guerreiro, José Brás, John Hardy, Andrew Singleton

**Affiliations:** 1 Department of Molecular Neuroscience and Reta Lila Weston Laboratories, UCL Institute of Neurology, London WC1N 1PJ, UK; 2 Laboratory of Neurogenetics, National Institute on Aging, Bethesda, MD 20892, USA

## Abstract

The development of next-generation sequencing technologies has allowed for the identification of several new genes and genetic factors in human genetics. Common results from the application of these technologies have revealed unexpected presentations for mutations in known disease genes. In this review, we summarize the major contributions of exome sequencing to the study of neurodegenerative disorders and other neurological conditions and discuss the interface between Mendelian and complex neurological diseases with a particular focus on pleiotropic events.

## INTRODUCTION

The development of new technologies has revolutionized the field of genetics. We are now able to determine variation and structure at a genome-wide level, with base-pair resolution and to assess its impact on phenotypes in an unprecedented manner.

Genome-wide association studies (GWAS) have been essential to uncover common variability contributing to various complex disorders. Whole-exome and whole-genome sequencing have identified rare variants causing or imparting large effects both on Mendelian as well as on complex diseases. But perhaps more interesting is how the integration of these technologies is uncovering some unexpected results when molecular data are associated with clinical phenotypes and when previously overlooked biological processes become central pathobiological pathways in a disease.

## TECHNOLOGICAL ADVANCES IN THE GENETICS OF DISEASE

Our understanding of the genetics underlying neurological disease has often been based on the development and application of new technologies. Perhaps the first example is how genetic linkage analyses of large pedigrees enabled the finding of several causative mutations underlying familial forms of disease. Case–control association studies on the other hand, which compared frequencies of genotypes in genes based on *a priori* biological hypotheses, did not have the same success for non-familial disorders. New loci for these common forms of disease have recently been found by GWAS where variation, distributed across the whole genome, is compared between thousands of cases and thousands of controls (1). For these forms of disease success clearly arrived when we were able to survey across the genome without the bias of biological plausibility. Thus, technological advances have allowed for the identification of very rare causative mutations underlying Mendelian forms of disease—through linkage analyses—and of common variants with low effects contributing to the susceptibility of late-onset and ‘sporadic’ disorders—through GWAS. However, limitations to these two approaches remain: linkage analyses are unable to adequately test variants that do not impart a very strong effect on disease, and GWAS is only suitable for variability that is relatively common in the general population. We are now able to test these types of variation by using whole-exome and other sequencing approaches (2,3).

## WE ARE GETTING WHAT WE PAID FOR

Exome sequencing is not only allowing for the quick identification of many genes as the cause of several diseases, it is also uncovering new risk factors for complex disorders. The application of this recently developed sequencing technology to neurological diseases is no exception and many examples of novel genes being associated with these disorders can now be found in the literature (Table 1).

**Table 1.** Examples of novel genes recently found to be associated with neurodegenerative diseases by the use of exome sequencing

Disease	Gene	Mutation(s)	Effect on disease	Refs.
Parkinson's disease	*VPS35*	Heterozygous p.Asp620Asn	Causative	(4,5)
Alzheimer's disease	*TREM2*	Heterozygous p.R47H	Increased risk (OR > 3)	(6,7)
Alzheimer's disease	*SORL1*	Heterozygous missense and nonsense	Potentially causative	(8)
Hereditary diffuse leukoencephalopathy with spheroids	*CSF1R*	Heterozygous missense, insertions and deletions all affecting the protein tyrosine kinase domain	Causative	(9)
Amyotrophic lateral sclerosis	*PFN1*	Heterozygous missense	Causative	(10)
Autosomal-recessive cerebellar ataxia with spasticity	*GBA2*	Homozygous missense and nonsense	Causative	(11)

Most causative novel genes have been identified in families where a specific phenotype segregates, either presenting a dominant or recessive pattern of inheritance. In the case of recessive diseases, the results obtained by exome sequencing are often guided by autozygosity mapping and in the case of dominant diseases, by genetic linkage analyses (12).

The number of findings is expected to increase as more samples; more funding and more collaborations are put in place. Several large projects are currently underway including several national and international collaborations. Probably, the largest of these projects is the Alzheimer's Disease Sequencing Project (ADSP - https://www.niagads.org/adsp/content/home), a joint effort between the National Human Genome Research Institute and the National Institute on Aging, which was one of the first projects launched under the $130 million National Alzheimer's Project, that started in 2012.

The democratization of massively parallel sequencing, and in particular of exome sequencing, has allowed for the identification (and will likely continue to identify) of an enormous number of novel genetic defects causing different diseases (Table 1). Even though the observation that mutations in the same gene can affect distinct clinical phenotypes has been a well-known and studied concept in molecular genetics (13), this avalanche of data resulting from the first era of exome sequencing has revealed novel connections between phenotypes that can help us to better understand the pathobiology of different neurological diseases. To consider these pleiotropic events, we will firstly discuss different instances where variants present in the same genes were found to be involved in different phenotypes and, secondly, we will focus on more specific cases where same-gene variants have been shown to exert different effects on diseases, depending on the way the variant is inherited.

## VARIANTS IN THE SAME GENES CAUSING DIFFERENT PHENOTYPES

The definition of ‘different phenotypes’ can be rather difficult. One illustrative example is the overlap between frontotemporal dementia (FTD) and amyotrophic lateral sclerosis: based on clinical, genetic and epidemiological data, these two previously thought of as completely independent entities, seem to be on opposite ends of a spectrum of disease, characterized pathologically by the presence of TDP-43 positive inclusions throughout the central nervous system (14–16). Adding to this clinical and neuropathological overlap is the recent molecular finding of hexanucleotide intronic expansions in *C9ORF72* as the cause of both FTD and ALS. It is currently not known why individuals with apparently the same genetic alteration develop either ALS or FTD (17,18). One possibility is that different expansion sizes are associated with different phenotypes; a hypothesis that has proved very difficult to test since the size of each mutated allele is difficult to assess.

Examples like the *C9ORF72* involvement in two clinically distinct entities raise questions about the best way to define diseases, particularly regarding the weight that should be put on the molecular findings and on the relationship between these findings and the neuropathological signatures associated with each disease. From the examples given in Table 2, it is difficult to reach definite conclusions for most cases: for *ATP13A2*, no pathological analysis of Kufor Rakeb brains has been performed yet; the Alzheimer's disease case with a CADASIL-associated mutation is still alive; and for *GRN* no pathological assessment of the NCL homozygous cases was done, although mice present with typical NCL lesions. However, for *VCP*, it has been clinically and neuropathologically demonstrated that mutations in this gene are responsible for cases diagnosed with either IBMPFD or ALS (19).

**Table 2.** Examples of mutations in the same genes causing different diseases, highlighting the role of NGS in uncovering pleiotropic events in neurodegenerative disorders

Gene	Initially described in disease(s)	Type of mutation	Also found in	Type of mutation	Refs.
ATP13A2*	Kufor Rakeb syndrome (KRS, OMIM #606693)	Frameshift homozygous	Neuronal ceroid-lipofuscinosis (NCL)	Missense homozygous	(20,21)
NOTCH3*	Cerebral autosomal dominant arteriopathy with subcortical infarcts and leukoencephalopathy (CADASIL, OMIM #125310)	Missense heterozygous changing a cysteine residue in the protein	Alzheimer's disease (AD, OMIM #104300)	Missense heterozygous changing a cysteine residue in the protein	(22,23)
*C9ORF72*	Frontotemporal dementia (FTD, OMIM #600274) and/or amyotrophic lateral sclerosis (ALS, OMIM #105400)	GGGGCC hexanucleotide intronic expansion			(17,18)
*SQSTM1*	Paget disease of the bone (PDB, OMIM #602080)	Missense heterozygous; frameshift; affecting splice site	FTD and ALS	Missense heterozygous; affecting splice site	(24–27)
GRN*	FTD	Heterozygous null	Neuronal ceroid lipofuscinosis (CLN11, OMIM #614706)	Homozygous null	(28–30)
VCP*	Inclusion body myopathy associated with Paget disease and frontotemporal dementia (IBMPFD, OMIM #167320)	Heterozygous missense	ALS and hereditary spastic paraplegia	Heterozygous missense	(19,31,32)
*PLA2G6*	Neurodegeneration with Brain Iron Accumulation 2A and 2B and Karak Syndrome (INAD, NBIA2A, OMIM # 256600 and NBIA2B, OMIM # 610217)	Missense, nonsense, splice-site, deletions, large intragenic deletions, homozygous or compound heterozygous	Adult-onset dystonia-parkinsonism (PARK14, OMIM 612953)	Homozygous and compound heterozygous missense and frameshift	(33,34)
*PSEN1*	AD	Missense, small insertions, heterozygous	Acne inversa (ACNINV3, OMIM #613737)	Frameshift deletion	(35,36)
*TRPV4*	Scapuloperoneal spinal muscular atrophy (SPSMA, OMIM #181405)	Missense heterozygous	Charcot-Marie-Tooth disease type 2C (HMSN2C, #606071)	Missense heterozygous	(37)
TPP1*	Late-infantile Neuronal Ceroid Lipofuscinosis 2 (CLN2, OMIM #204500)	Missense, nonsense, splice-site affecting, deletions, insertions and deletion–insertion homozygous or compound heterozygous	Autosomal recessive spinocerebellar ataxia 7 (SCAR7, OMIM %609270)	Compound heterozygous	(38–40)

*Second association found by exome sequencing. Refs., references associated with the original descriptions in the different diseases.

In line with the aforementioned spectrum of FTD-ALS disease, these results expand even more the range of overlap by including an association of ALS with bone dysfunction and myopathy. At the same time, these results point towards the involvement of cellular protein degradation processes in the molecular patholog of ALS. The involvement of such pathobiological pathways in ALS can also be substantiated by the recent findings of *SQSTM1* (encoding the p62 protein, a multifunctional protein that binds ubiquitin and is one of the best-known autophagic substrates) (41) mutations both in ALS and FTD (24,25).

Many other atypical phenotypical presentations are suggested in the literature, particularly for different dementias, for example *PSEN1* mutations were found in FTD cases (42,43) and a nonsense mutation in *PRNP* was associated with clinical and neuropathological features of AD (44).

Although these overlaps between different clinical entities can generate difficulties in the establishment of definitive diagnoses, the assessment of mixed cohorts has also revealed interesting genetic findings: high throughput deep sequencing identified several *GRN* and *MAPT* mutations in AD clinical cohorts (possibly due to misdiagnoses) (45), a genome-wide analysis uncovered several risk loci with shared effects on five psychiatric disorders (46) and the joint analysis of multisystem proteinopathy cases reflecting the expanded phenotype and proteinaceous pathology characterizing diseases as IBM, FTD, ALS and PDB has allowed for the identification of causative mutations in *hnRNPA2B1* and *hnRNPA1* (47).

It is also possible that the relative abundance of pleiotropic effects being found by exome sequencing results from the difficulty in interpreting the pathogenic impact of some variants identified through NGS. High throughput techniques have shown that each individual carries a large number of genomic variations of unclear significance and for some of these variations, especially if found in isolated patients and in the absence of functional studies, the establishment of their pathogenicity in relation to a specific phenotype may be extremely problematic. As more and more samples are being sequenced, we see that variants previously thought to be pathogenic are being found in healthy individuals, challenging the definition of causality and the interpretation of NGS results.

The establishment of pathogenicity to variants identified through NGS is far from being a straightforward process, and it is currently posing as a potential confounder for the identification of true pleiotropic events and requiring new tools to adequately assess this issue.

## VARIANTS IN THE SAME GENE CAN CAUSE A RARE EARLY-ONSET SEVERE DISEASE AND MODULATE THE RISK FOR A LATE-ONSET COMMON DISEASE

One result we previously predicted is the finding of pairs of diseases previously thought to be unrelated and that are influenced by different types of genetic variation in the same gene (48): one disease is usually severe, has an early onset, and is caused by homozygous loss-of-function mutations, while the other is a late-onset disease with increased susceptibility caused by heterozygous (probably with partial loss-of-function) variants in the same gene (Table 3).

**Table 3.** Examples of homozygous/heterozygous variants in the same gene causing a severe early-onset disease and increasing the risk for a different late-onset disease

Gene	Homozygous mutations cause	Refs.	Heterozygous variants increase risk for	Refs.
*TREM2*	Nasu-Hakola and FTD-like syndrome without bone disease	(49,50)	Alzheimer's disease	(6,7)
*GBA*	Gaucher's disease	(51)	Parkinson's disease	(52)

Refs., references.

Usually mutations in one gene cause specific phenotypes either in the heterozygous or in the homozygous state, but generally not in both. In autosomal recessive diseases, heterozygous individuals are usually healthy. In autosomal dominant disorders, the allele frequency for the mutation is low, thus homozygous individuals are very rare, with the exception of highly inbred populations. When observed, these homozygous cases are usually very similar to the heterozygous affected family members [Huntington disease (53), Parkinson's disease (54), Creutzfeldt-Jakob disease (55)] or have a more severe form of the same phenotype (Spinocerebellar Ataxia-2, -3 and -6, for example) (56–59).

The occurrence in the same locus of genetic variation with different modes of inheritance imparting different effects on different diseases can partially be explained by natural selection: homozygous loss of function mutations cause early-onset disorders and many individuals with these mutations die before reaching reproductive age, contributing to the rare frequency of the disease and of the mutations. On the other hand, heterozygous variants confer risk to a disorder with an onset usually occurring beyond reproductive age, making these variants and diseases more common in the population.

Homozygous mutations in *TREM2* were originally found to be the cause of Nasu-Hakola disease (also known as polycystic lipomembranous osteodysplasia with sclerosing leukoencephalopathy), a rare autosomal recessive form of dementia presenting with pain and swelling of wrists and/or ankles due to bone cysts and usually followed by bone fractures (49). Patients usually die in the fourth decade of life presenting the later features of the disease that resemble those of AD or FTD. The same type of mutations (and in some cases the same exact mutations) have been described in patients presenting with frontotemporal dementia, but with no associated bone phenotypes (50,60). More recently, a rare heterozygous variant (p.R47H) was associated with an increased risk of AD (6,7). TREM2 is a membrane protein that forms a receptor–signalling complex with TYROBP (also known as DAP-12) and works to activate immune responses in different cells from the myeloid lineage, like macrophages and dendritic cells. It is thought to have an anti-inflammatory role in the brain (49). In AD, loss-of-function or partial loss-of-function mutations in the gene are expected to alter inflammatory processes and lead to a decreased ability of clearing amyloid plaques, with a consequent increase in cell death and cognitive decline. Common variation in the *TREM2* locus has also been associated by GWAS with C-reactive protein levels (61) and potential associations of p.R47H with FTD, ALS and Parkinson's disease have also been reported (62–65), suggesting a potential role for TREM2 across different neurodegenerative disorders.

In a similar fashion, homozygous mutations in the gene coding for the glucocerebrosidase (GBA) enzyme cause Gaucher's disease, a lysosomal storage disease characterized by the accumulation of GBAs (51), while heterozygous variants in *GBA* have been associated with an increased risk of PD (52), DLB (66) and PD with dementia (67).

These findings have confirmed the central role of inflammation and lysosomal pathways in AD and PD, respectively.

Also interesting to note are the associations between heterozygous variants in autosomal recessive PD loci and different pathologies. Homozygous mutations in *PARK2* (encoding parkin, an E3 ubiquitin ligase) are known to cause early-onset forms of Parkinson disease (68) and the association of heterozygous variants with an increased risk of PD has long been debated. More recently, *PARK2* somatic mutations have been associated with different types of cancer (69), suggesting that germline mutations in *PARK2* cause PD and somatic mutations contribute to cancer (for a review see Plun-Favreau *et al.*) (70). Additionally, genome-wide analysis of rare copy number variants identified *PARK2* as a candidate gene for attention-deficit/hyperactivity disorder and GWAS have found significant associations between common variability in this locus with lumbar disc degeneration (in a meta-analysis of northern Europeans) (71), ageing (by performing linkage and association in large Amish kindreds) (72), pancreatic cancer in the Japanese population (73) and metabolite levels (74). The fact that *PARK2* is embedded in a common fragile site (FRA6R) and, consequently, is particularly prone to breaks, may explain the frequent occurrence of *PARK2* gross mutations like deletions in cancer cells and the association of copy number variants with attention-deficit/hyperactivity disorder (75). However, it is difficult to anticipate a related mechanism for the associations established with common point variability in the locus, especially since similar associations have been identified for other PARK loci: by GWAS, *PARK7* has been associated with ulcerative colitis (76) and celiac disease (77), while *PLA2G6* has been associated with susceptibility to melanoma (78) and cutaneous nevi (79,80) (high melanocytic nevi count is the strongest known risk factor for cutaneous melanoma). Interestingly, *LRRK2* has also been associated with inflammatory bowel disease, Crohn's disease and leprosy (81–84).

## POSSIBLE MECHANISMS FOR COMPLEX ASSOCIATIONS BETWEEN MOLECULAR FINDINGS AND PHENOTYPES

While revealing this increasing complexity (that can be considered to challenge the basis of Mendelian genetics), NGS techniques are also providing some relevant insights into the mechanisms underlying such complexity. One example previously mentioned is the possibility that different expansion sizes of *C9ORF72* are related to either a phenotype of FTD or ALS. However, besides this more obvious possible correlation between different types of mutations and distinct phenotypes, other mechanisms can be involved and these include: oligogenic or polygenic inheritance, variants in distinct genes acting as phenotypic modifiers of a monogenic disorder, different genetic background, gene–gene interactions, differential expression levels in different cell types, environmental factors or epigenetic effects. All of which will require much larger and deeply studied data sets to be tested on.

## CONCLUSION

Results from exome sequencing analyses have reinforced the notion that some neurodegenerative diseases are part of pathological spectrums arising from common molecular processes. In the context of late-onset diseases, and in particular diseases with a long preclinical phase, it is not surprising that there is substantial clinical heterogeneity given the potential for influence of different factors (including genetic and environmental) from the inception of the process to phenotypic presentation. This would be more obvious if the genes mutated were involved with repair or response to an insult—the end point would be determined by what the initial problem was and where it started.

These commonalities observed between different diseases cannot only be seen in the form of pure pleiotropic genetic effects but also, and perhaps more interestingly, when variants in the same gene but with different patterns of inheritance cause a severe early-onset disease and modulate the risk for a more common and less severe late-onset disorder.

Although extraordinary advances are being made by the application of exome sequencing to the study of neurological diseases, it is also important to mention that at least part of the genetic lesions contributing to these diseases will not be amenable to be found by exome or even genome sequencing. The large intronic hexanucleotide expansion in *C9ORF72* is a clear example in neurological diseases but examples from other diseases are also arising, like the large VNTR in *MUC1* causing medullary cystic kidney disease type 1 (85).

Clearly some of these results will help clinicians understand the co-occurrence of clinical phenotypes in patients, as well as point to wider genetic screens when the obvious candidate genes are negative for mutations, and to potential druggable targets. The latter is already evident from the recent programmes that several drug companies have started on inflammation and associated processes for Alzheimer's disease, following the identification of TREM2 as a risk gene for this disorder.

In summary, results showing genetically overlapping diseases have clear implications for the clinical diagnoses and follow-up of patients, but are also of great importance to uncover the molecular mechanisms underlying these pathologies.

*Conflict of Interest statement*. None declared.

## FUNDING

The authors' work is supported by the Alzheimer's Research UK and by the Wellcome Trust/MRC Joint Call in Neurodegeneration award (WT089698) to the UK Parkinson's Disease Consortium (UKPDC) whose members are from the UCL/Institute of Neurology, the University of Sheffield and the MRC Protein Phosphorylation Unit at the University of Dundee. Funding to pay the Open Access publication charges for this article was provided by the Wellcome Trust/MRC.
